# 10-(1,3-Benzothia­zol-2-yl)-1,1,7,7-tetra­methyl-2,3,6,7-tetra­hydro-1*H*,5*H*,11*H*-pyrano[3,2-*g*]pyrido[3,2,1-*hi*]quinoline

**DOI:** 10.1107/S1600536810011086

**Published:** 2010-03-27

**Authors:** Ki-Min Park, Youngjin Kang

**Affiliations:** a Department of Chemistry & Research Institute of Natural Science, Gyeongsang National University, Jinju 660-701, Republic of Korea; b Division of Science Education, Kangwon National University, Chuncheon 200-701, Republic of Korea

## Abstract

In the title compound, C_26_H_26_N_2_O_2_S, the dihedral angle between the benzothia­zole and coumarin rings is 8.34 (7)°, indicating that the overall benzothia­zole substituent is almost coplanar with the coumarin rings. An intra­molecular S⋯O [2.813 (1) Å] contact may help to stabilize the mol­ecular conformation. In the crystal structure, π–π stacking inter­actions [centroid–centroid distances = 3.480 (2) Å] link pairs of mol­ecules.

## Related literature

For background to organic light-emitting diodes (OLEDs), see: Lee *et al.* (2009 ▶). For the use of the title compound as an organic light-emitting diode, see: White *et al.*(2010 ▶). For S⋯O inter­actions, see: Mellor *et al.* (1971 ▶); Kucsman *et al.* (1984 ▶). For the crystal structure of benzothia­zole-ethyl­coumarin, see: Padilla-Martínez *et al.* (2003 ▶) and for that of coumarin, see: Gavuzzo *et al.* (1974 ▶); Chinnakali *et al.* (1999 ▶).
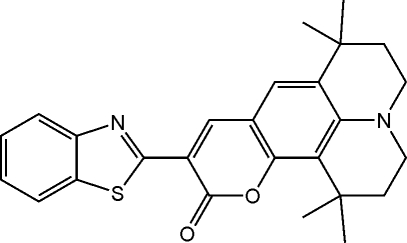


## Experimental

### 

#### Crystal data

C_26_H_26_N_2_O_2_S
*M* *_r_* = 430.55
Monoclinic, 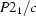

*a* = 9.2180 (4) Å
*b* = 13.7079 (6) Å
*c* = 18.6885 (6) Å
β = 115.890 (2)°
*V* = 2124.46 (15) Å^3^
*Z* = 4
Mo *K*α radiation
μ = 0.18 mm^−1^
*T* = 173 K
0.50 × 0.40 × 0.40 mm

#### Data collection

Bruker SMART CCD area-detector diffractometer
11820 measured reflections
4179 independent reflections
3592 reflections with *I* > 2σ(*I*)
*R*_int_ = 0.030

#### Refinement

*R*[*F* ^2^ > 2σ(*F* ^2^)] = 0.036
*wR*(*F* ^2^) = 0.100
*S* = 1.06
4179 reflections
280 parameters
H-atom parameters constrained
Δρ_max_ = 0.31 e Å^−3^
Δρ_min_ = −0.28 e Å^−3^

### 

Data collection: *SMART* (Bruker, 2000 ▶); cell refinement: *SAINT-Plus* (Bruker, 2000 ▶); data reduction: *SAINT-Plus*; program(s) used to solve structure: *SHELXS97* (Sheldrick, 2008 ▶); program(s) used to refine structure: *SHELXL97* (Sheldrick, 2008 ▶); molecular graphics: *SHELXTL* (Sheldrick, 2008 ▶); software used to prepare material for publication: *SHELXTL*.

## Supplementary Material

Crystal structure: contains datablocks I, global. DOI: 10.1107/S1600536810011086/sj2754sup1.cif

Structure factors: contains datablocks I. DOI: 10.1107/S1600536810011086/sj2754Isup2.hkl

Additional supplementary materials:  crystallographic information; 3D view; checkCIF report

## Figures and Tables

**Fig. 1.**
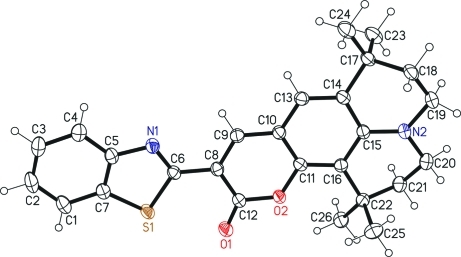
The molecular structure of title compound, showing the atom-numbering scheme. Displacement ellipsoids are drawn at the 50% probability level; hydrogen atoms are drawn as spheres of arbitrary radius.

**Fig. 2.**
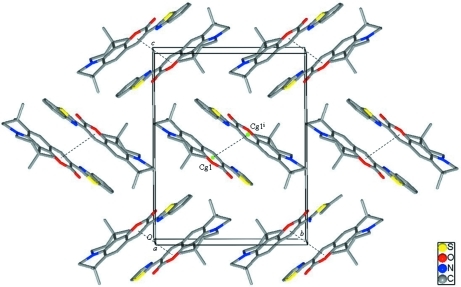
π–π interactions (dotted lines) in the title compound. Cg denotes the O2, C8–C12 ring centroid. [Symmetry codes: (i) -x+1,-y+1,-z+1.]

## supplementary crystallographic information

### Comment

Luminescent compounds have attracted much attention owing to their varied applications, such as in photonics and as organic light-emitting diodes (Lee *et al.*,  2009). Among such luminescent compounds, 10-(2-Benzothiazolyl)-1,1,7,7-tetramethyl-2,3,6,7-tetrahydro-1H,5H,11H- benzo[l]pyr ano[6,7,8-ij] quinolizin-11-one, often referred as C545T, is regarded as an excellent fluorescent compound and has been widely studied because of its ability to achieve high external quantum efficiency in organic light-emitting diodes (White *et al.*,  2010).  Therefore, as a good emitter the structure of C545T is of interest to materials chemists.

In the title compound (Scheme 1, Fig.1), the benzothiazole and coumarin segments lie in the same plane with a dihedral angle of 8.34 (7)° between the respective planes. This coplanarity may be assisted by a short intramolecular contact (2.813 (1) Å) between S1 and O1 (Mellor *et al.*, 1971; Kucsman *et al.*, 1984). All bond lengths and bond angles are normal and comparable to those of observed in the structures of coumarin and benzothiazole derivatives (Gavuzzo *et al.*, 1974; Chinnakali *et al.*, 1999; Padilla-Martínez *et al.*, 2003).

A π—π stacking interaction is observed between two adjacent coumarin segments in the crystal packing is observed [C12···C9^i^ = 3.480 (2) Å; Cg1···Cg1^i^ = 3.778 Å; where Cg1 is the centroid of the O2, C8–C12 ring; symmetry code (i) 1-x, 1-y, 1-z] (Fig. 2).

### Experimental

10-(2-Benzothiazolyl)-1,1,7,7-tetramethyl- 2,3,6,7-tetrahydro-1*H*,5*H*,11*H*-benzo[*l*] pyrano[6,7,8-*ij*]quinolizin-11-one (C545T) was purchased from the Aldrich Chemical Company. Slow evaporation of a solution of CH_2_Cl_2_ and hexane (1:1, v:v) gave suitable single crystals for X-ray analysis.

### Refinement

All H-atoms were positioned geometrically and refined using a riding model with d(C—H) = 0.95 Å, *U*_iso_ =1.2*U*_eq_(C) for aromatic, 0.99 Å, *U*_iso_ = 1.2*U*_eq_(C) for CH_2_, and 0.98 Å, *U*_iso_ = 1.5*U*_eq_(C) for CH_3_ atoms.

### Crystal data

**Table:**

C_26_H_26_N_2_O_2_S	*F*(000) = 912
*M**_r_* = 430.55	*D*_x_ = 1.346 Mg m^−^^3^
Monoclinic, *P*2_1_/*c*	Mo *K*α radiation, λ = 0.71073 Å
Hall symbol: -P 2ybc	Cell parameters from 7330 reflections
*a* = 9.2180 (4) Å	θ = 2.4–28.3°
*b* = 13.7079 (6) Å	µ = 0.18 mm^−^^1^
*c* = 18.6885 (6) Å	*T* = 173 K
β = 115.890 (2)°	Block, orange
*V* = 2124.46 (15) Å^3^	0.50 × 0.40 × 0.40 mm
*Z* = 4	

### Data collection

**Table:**

Bruker SMART CCD area-detector diffractometer	3592 reflections with *I* > 2σ(*I*)
Radiation source: fine-focus sealed tube	*R*_int_ = 0.030
graphite	θ_max_ = 26.0°, θ_min_ = 1.9°
φ and ω scans	*h* = −11→9
11820 measured reflections	*k* = −12→16
4179 independent reflections	*l* = −20→23

### Refinement

**Table:**

Refinement on *F*^2^	Primary atom site location: structure-invariant direct methods
Least-squares matrix: full	Secondary atom site location: difference Fourier map
*R*[*F*^2^ > 2σ(*F*^2^)] = 0.036	Hydrogen site location: inferred from neighbouring sites
*wR*(*F*^2^) = 0.100	H-atom parameters constrained
*S* = 1.06	*w* = 1/[σ^2^(*F*_o_^2^) + (0.044*P*)^2^ + 1.0847*P*] where *P* = (*F*_o_^2^ + 2*F*_c_^2^)/3
4179 reflections	(Δ/σ)_max_ = 0.001
280 parameters	Δρ_max_ = 0.31 e Å^−^^3^
0 restraints	Δρ_min_ = −0.28 e Å^−^^3^

### Special details

**Table:**

Geometry. All esds (except the esd in the dihedral angle between two l.s. planes) are estimated using the full covariance matrix. The cell esds are taken into account individually in the estimation of esds in distances, angles and torsion angles; correlations between esds in cell parameters are only used when they are defined by crystal symmetry. An approximate (isotropic) treatment of cell esds is used for estimating esds involving l.s. planes.
Refinement. Refinement of *F*^2^ against ALL reflections. The weighted *R*-factor wR and goodness of fit *S* are based on *F*^2^, conventional *R*-factors *R* are based on *F*, with *F* set to zero for negative *F*^2^. The threshold expression of *F*^2^ > σ(*F*^2^) is used only for calculating *R*-factors(gt) etc. and is not relevant to the choice of reflections for refinement. *R*-factors based on *F*^2^ are statistically about twice as large as those based on *F*, and *R*- factors based on ALL data will be even larger.

### Fractional atomic coordinates and isotropic or equivalent isotropic displacement parameters (Å^2^)

**Table:**

	*x*	*y*	*z*	*U*_iso_*/*U*_eq_	
S1	0.19362 (5)	0.61269 (3)	0.29844 (2)	0.02617 (12)	
O1	0.48027 (14)	0.50800 (9)	0.33612 (7)	0.0319 (3)	
O2	0.57724 (12)	0.38158 (8)	0.41606 (6)	0.0239 (2)	
N1	0.06477 (15)	0.51630 (9)	0.37615 (8)	0.0241 (3)	
N2	0.79705 (17)	0.09754 (10)	0.57717 (8)	0.0288 (3)	
C1	−0.0975 (2)	0.72222 (13)	0.23952 (10)	0.0329 (4)	
H1	−0.0649	0.7633	0.2083	0.039*	
C2	−0.2464 (2)	0.73381 (13)	0.23991 (11)	0.0362 (4)	
H2	−0.3164	0.7841	0.2088	0.043*	
C3	−0.2958 (2)	0.67300 (13)	0.28511 (10)	0.0311 (4)	
H3	−0.3993	0.6820	0.2836	0.037*	
C4	−0.19676 (19)	0.60012 (12)	0.33190 (10)	0.0272 (3)	
H4	−0.2305	0.5594	0.3630	0.033*	
C5	−0.04516 (18)	0.58726 (11)	0.33269 (9)	0.0236 (3)	
C6	0.19373 (18)	0.52131 (11)	0.36444 (9)	0.0214 (3)	
C7	0.00332 (19)	0.64804 (12)	0.28657 (9)	0.0252 (3)	
C8	0.32875 (18)	0.45412 (11)	0.40379 (9)	0.0217 (3)	
C9	0.32966 (18)	0.38973 (11)	0.45990 (9)	0.0232 (3)	
H9	0.2454	0.3929	0.4761	0.028*	
C10	0.45101 (18)	0.31905 (11)	0.49446 (9)	0.0218 (3)	
C11	0.57626 (18)	0.31438 (11)	0.47106 (9)	0.0210 (3)	
C12	0.46105 (18)	0.45318 (11)	0.38203 (9)	0.0228 (3)	
C13	0.44984 (18)	0.24849 (11)	0.54910 (9)	0.0233 (3)	
H13	0.3675	0.2512	0.5666	0.028*	
C14	0.56330 (18)	0.17633 (11)	0.57769 (9)	0.0219 (3)	
C15	0.68816 (18)	0.17229 (11)	0.55131 (9)	0.0219 (3)	
C16	0.69810 (18)	0.24492 (11)	0.49835 (9)	0.0214 (3)	
C17	0.56258 (19)	0.10207 (11)	0.63862 (9)	0.0243 (3)	
C18	0.6185 (2)	0.00427 (12)	0.61992 (10)	0.0316 (4)	
H18A	0.6225	−0.0449	0.6596	0.038*	
H18B	0.5397	−0.0184	0.5668	0.038*	
C19	0.7820 (3)	0.01276 (14)	0.62120 (12)	0.0402 (5)	
H19A	0.8645	0.0174	0.6771	0.048*	
H19B	0.8040	−0.0472	0.5980	0.048*	
C20	0.9368 (2)	0.09144 (14)	0.56085 (11)	0.0352 (4)	
H20A	0.9156	0.0432	0.5180	0.042*	
H20B	1.0305	0.0683	0.6091	0.042*	
C21	0.9768 (2)	0.18838 (13)	0.53606 (10)	0.0312 (4)	
H21A	1.0613	0.1787	0.5176	0.037*	
H21B	1.0210	0.2322	0.5828	0.037*	
C22	0.82998 (18)	0.23758 (11)	0.46957 (9)	0.0237 (3)	
C23	0.6774 (2)	0.13677 (13)	0.72253 (10)	0.0342 (4)	
H23A	0.7856	0.1461	0.7258	0.051*	
H23B	0.6817	0.0877	0.7616	0.051*	
H23C	0.6383	0.1987	0.7338	0.051*	
C24	0.3950 (2)	0.08820 (13)	0.63537 (11)	0.0342 (4)	
H24A	0.3197	0.0660	0.5821	0.051*	
H24B	0.3572	0.1503	0.6471	0.051*	
H24D	0.4006	0.0394	0.6748	0.051*	
C25	0.7662 (2)	0.17553 (14)	0.39363 (10)	0.0357 (4)	
H25A	0.6727	0.2079	0.3519	0.054*	
H25D	0.7340	0.1112	0.4046	0.054*	
H25B	0.8510	0.1678	0.3758	0.054*	
C26	0.8916 (2)	0.33538 (12)	0.45338 (10)	0.0302 (4)	
H26D	0.8026	0.3703	0.4111	0.045*	
H26A	0.9772	0.3233	0.4367	0.045*	
H26B	0.9345	0.3749	0.5020	0.045*	

### Atomic displacement parameters (Å^2^)

**Table:**

	*U*^11^	*U*^22^	*U*^33^	*U*^12^	*U*^13^	*U*^23^
S1	0.0285 (2)	0.0226 (2)	0.0269 (2)	0.00232 (16)	0.01166 (17)	0.00763 (15)
O1	0.0331 (6)	0.0313 (6)	0.0365 (6)	0.0063 (5)	0.0199 (5)	0.0135 (5)
O2	0.0244 (5)	0.0235 (6)	0.0255 (5)	0.0046 (4)	0.0126 (5)	0.0070 (4)
N1	0.0239 (7)	0.0215 (7)	0.0263 (7)	0.0020 (5)	0.0104 (5)	0.0026 (5)
N2	0.0340 (8)	0.0252 (7)	0.0295 (7)	0.0102 (6)	0.0158 (6)	0.0076 (6)
C1	0.0364 (9)	0.0253 (8)	0.0299 (9)	0.0046 (7)	0.0079 (7)	0.0064 (7)
C2	0.0345 (9)	0.0279 (9)	0.0331 (9)	0.0109 (7)	0.0027 (7)	0.0026 (7)
C3	0.0247 (8)	0.0291 (9)	0.0316 (9)	0.0057 (7)	0.0048 (7)	−0.0065 (7)
C4	0.0255 (8)	0.0239 (8)	0.0289 (8)	0.0000 (6)	0.0088 (7)	−0.0042 (6)
C5	0.0256 (8)	0.0182 (7)	0.0221 (7)	0.0015 (6)	0.0059 (6)	−0.0019 (6)
C6	0.0254 (8)	0.0171 (7)	0.0206 (7)	−0.0004 (6)	0.0090 (6)	0.0008 (6)
C7	0.0262 (8)	0.0218 (8)	0.0234 (7)	0.0007 (6)	0.0072 (6)	−0.0017 (6)
C8	0.0227 (7)	0.0189 (7)	0.0225 (7)	0.0002 (6)	0.0089 (6)	0.0004 (6)
C9	0.0223 (7)	0.0233 (8)	0.0249 (8)	−0.0001 (6)	0.0111 (6)	0.0011 (6)
C10	0.0224 (7)	0.0209 (7)	0.0222 (7)	0.0006 (6)	0.0097 (6)	0.0017 (6)
C11	0.0240 (7)	0.0192 (7)	0.0183 (7)	−0.0015 (6)	0.0077 (6)	0.0004 (6)
C12	0.0236 (8)	0.0211 (8)	0.0218 (7)	0.0005 (6)	0.0083 (6)	0.0018 (6)
C13	0.0242 (8)	0.0241 (8)	0.0228 (7)	−0.0005 (6)	0.0114 (6)	0.0015 (6)
C14	0.0261 (8)	0.0191 (7)	0.0191 (7)	−0.0014 (6)	0.0085 (6)	−0.0001 (6)
C15	0.0248 (8)	0.0193 (7)	0.0182 (7)	0.0015 (6)	0.0062 (6)	−0.0011 (6)
C16	0.0222 (7)	0.0210 (7)	0.0190 (7)	−0.0001 (6)	0.0073 (6)	−0.0020 (6)
C17	0.0310 (8)	0.0207 (8)	0.0204 (7)	0.0005 (6)	0.0104 (6)	0.0029 (6)
C18	0.0489 (11)	0.0204 (8)	0.0282 (8)	0.0052 (7)	0.0195 (8)	0.0059 (7)
C19	0.0569 (12)	0.0296 (9)	0.0432 (10)	0.0196 (9)	0.0304 (9)	0.0164 (8)
C20	0.0344 (9)	0.0367 (10)	0.0380 (10)	0.0154 (8)	0.0190 (8)	0.0092 (8)
C21	0.0263 (8)	0.0351 (9)	0.0316 (9)	0.0071 (7)	0.0121 (7)	0.0031 (7)
C22	0.0238 (8)	0.0244 (8)	0.0230 (7)	0.0019 (6)	0.0102 (6)	−0.0004 (6)
C23	0.0467 (10)	0.0284 (9)	0.0219 (8)	−0.0023 (8)	0.0098 (7)	0.0022 (7)
C24	0.0395 (10)	0.0278 (9)	0.0390 (10)	−0.0006 (8)	0.0205 (8)	0.0098 (7)
C25	0.0412 (10)	0.0386 (10)	0.0301 (9)	−0.0010 (8)	0.0181 (8)	−0.0075 (8)
C26	0.0242 (8)	0.0319 (9)	0.0359 (9)	−0.0005 (7)	0.0144 (7)	0.0033 (7)

### Geometric parameters (Å, °)

**Table:**

S1—C7	1.7382 (16)	C14—C17	1.530 (2)
S1—C6	1.7576 (15)	C15—C16	1.435 (2)
O1—C12	1.2104 (18)	C16—C22	1.532 (2)
O2—C11	1.3832 (18)	C17—C18	1.530 (2)
O2—C12	1.3857 (18)	C17—C24	1.531 (2)
N1—C6	1.301 (2)	C17—C23	1.535 (2)
N1—C5	1.383 (2)	C18—C19	1.501 (3)
N2—C15	1.367 (2)	C18—H18A	0.9900
N2—C20	1.450 (2)	C18—H18B	0.9900
N2—C19	1.465 (2)	C19—H19A	0.9900
C1—C2	1.385 (3)	C19—H19B	0.9900
C1—C7	1.399 (2)	C20—C21	1.505 (3)
C1—H1	0.9500	C20—H20A	0.9900
C2—C3	1.397 (3)	C20—H20B	0.9900
C2—H2	0.9500	C21—C22	1.537 (2)
C3—C4	1.378 (2)	C21—H21A	0.9900
C3—H3	0.9500	C21—H21B	0.9900
C4—C5	1.402 (2)	C22—C25	1.535 (2)
C4—H4	0.9500	C22—C26	1.537 (2)
C5—C7	1.405 (2)	C23—H23A	0.9800
C6—C8	1.463 (2)	C23—H23B	0.9800
C8—C9	1.368 (2)	C23—H23C	0.9800
C8—C12	1.444 (2)	C24—H24A	0.9800
C9—C10	1.406 (2)	C24—H24B	0.9800
C9—H9	0.9500	C24—H24D	0.9800
C10—C11	1.403 (2)	C25—H25A	0.9800
C10—C13	1.410 (2)	C25—H25D	0.9800
C11—C16	1.389 (2)	C25—H25B	0.9800
C13—C14	1.367 (2)	C26—H26D	0.9800
C13—H13	0.9500	C26—H26A	0.9800
C14—C15	1.438 (2)	C26—H26B	0.9800
			
C7—S1—C6	88.83 (7)	C14—C17—C23	109.21 (13)
C11—O2—C12	123.93 (12)	C18—C17—C23	110.73 (14)
C6—N1—C5	110.66 (13)	C24—C17—C23	108.23 (14)
C15—N2—C20	123.48 (14)	C19—C18—C17	111.36 (14)
C15—N2—C19	123.93 (14)	C19—C18—H18A	109.4
C20—N2—C19	112.54 (13)	C17—C18—H18A	109.4
C2—C1—C7	117.92 (16)	C19—C18—H18B	109.4
C2—C1—H1	121.0	C17—C18—H18B	109.4
C7—C1—H1	121.0	H18A—C18—H18B	108.0
C1—C2—C3	121.35 (16)	N2—C19—C18	113.16 (14)
C1—C2—H2	119.3	N2—C19—H19A	108.9
C3—C2—H2	119.3	C18—C19—H19A	108.9
C4—C3—C2	121.03 (16)	N2—C19—H19B	108.9
C4—C3—H3	119.5	C18—C19—H19B	108.9
C2—C3—H3	119.5	H19A—C19—H19B	107.8
C3—C4—C5	118.62 (16)	N2—C20—C21	111.93 (14)
C3—C4—H4	120.7	N2—C20—H20A	109.2
C5—C4—H4	120.7	C21—C20—H20A	109.2
N1—C5—C4	124.45 (14)	N2—C20—H20B	109.2
N1—C5—C7	115.44 (14)	C21—C20—H20B	109.2
C4—C5—C7	120.11 (14)	H20A—C20—H20B	107.9
N1—C6—C8	121.43 (13)	C20—C21—C22	112.88 (14)
N1—C6—S1	115.79 (11)	C20—C21—H21A	109.0
C8—C6—S1	122.77 (11)	C22—C21—H21A	109.0
C1—C7—C5	120.97 (15)	C20—C21—H21B	109.0
C1—C7—S1	129.75 (14)	C22—C21—H21B	109.0
C5—C7—S1	109.27 (11)	H21A—C21—H21B	107.8
C9—C8—C12	119.13 (14)	C16—C22—C25	108.57 (13)
C9—C8—C6	120.73 (14)	C16—C22—C21	107.36 (12)
C12—C8—C6	120.12 (13)	C25—C22—C21	110.56 (14)
C8—C9—C10	122.29 (14)	C16—C22—C26	115.48 (13)
C8—C9—H9	118.9	C25—C22—C26	109.08 (13)
C10—C9—H9	118.9	C21—C22—C26	105.74 (13)
C11—C10—C9	119.09 (14)	C17—C23—H23A	109.5
C11—C10—C13	117.51 (14)	C17—C23—H23B	109.5
C9—C10—C13	123.32 (14)	H23A—C23—H23B	109.5
O2—C11—C16	117.57 (13)	C17—C23—H23C	109.5
O2—C11—C10	118.32 (13)	H23A—C23—H23C	109.5
C16—C11—C10	124.09 (14)	H23B—C23—H23C	109.5
O1—C12—O2	116.23 (13)	C17—C24—H24A	109.5
O1—C12—C8	126.66 (14)	C17—C24—H24B	109.5
O2—C12—C8	117.11 (13)	H24A—C24—H24B	109.5
C14—C13—C10	122.20 (14)	C17—C24—H24D	109.5
C14—C13—H13	118.9	H24A—C24—H24D	109.5
C10—C13—H13	118.9	H24B—C24—H24D	109.5
C13—C14—C15	118.95 (14)	C22—C25—H25A	109.5
C13—C14—C17	121.41 (14)	C22—C25—H25D	109.5
C15—C14—C17	119.61 (13)	H25A—C25—H25D	109.5
N2—C15—C16	120.40 (14)	C22—C25—H25B	109.5
N2—C15—C14	118.82 (14)	H25A—C25—H25B	109.5
C16—C15—C14	120.78 (13)	H25D—C25—H25B	109.5
C11—C16—C15	116.37 (13)	C22—C26—H26D	109.5
C11—C16—C22	123.68 (13)	C22—C26—H26A	109.5
C15—C16—C22	119.74 (13)	H26D—C26—H26A	109.5
C14—C17—C18	107.56 (12)	C22—C26—H26B	109.5
C14—C17—C24	112.67 (13)	H26D—C26—H26B	109.5
C18—C17—C24	108.46 (14)	H26A—C26—H26B	109.5
			
C7—C1—C2—C3	−0.6 (3)	C10—C13—C14—C17	178.36 (14)
C1—C2—C3—C4	1.0 (3)	C20—N2—C15—C16	6.7 (2)
C2—C3—C4—C5	−0.7 (2)	C19—N2—C15—C16	−170.41 (16)
C6—N1—C5—C4	179.25 (15)	C20—N2—C15—C14	−173.69 (15)
C6—N1—C5—C7	−0.07 (19)	C19—N2—C15—C14	9.2 (2)
C3—C4—C5—N1	−179.17 (15)	C13—C14—C15—N2	−177.07 (14)
C3—C4—C5—C7	0.1 (2)	C17—C14—C15—N2	4.7 (2)
C5—N1—C6—C8	179.91 (13)	C13—C14—C15—C16	2.5 (2)
C5—N1—C6—S1	−0.49 (17)	C17—C14—C15—C16	−175.77 (13)
C7—S1—C6—N1	0.69 (13)	O2—C11—C16—C15	−176.04 (12)
C7—S1—C6—C8	−179.71 (13)	C10—C11—C16—C15	2.5 (2)
C2—C1—C7—C5	0.0 (2)	O2—C11—C16—C22	−1.4 (2)
C2—C1—C7—S1	178.80 (13)	C10—C11—C16—C22	177.11 (14)
N1—C5—C7—C1	179.56 (14)	N2—C15—C16—C11	175.88 (14)
C4—C5—C7—C1	0.2 (2)	C14—C15—C16—C11	−3.7 (2)
N1—C5—C7—S1	0.57 (17)	N2—C15—C16—C22	1.0 (2)
C4—C5—C7—S1	−178.78 (12)	C14—C15—C16—C22	−178.56 (13)
C6—S1—C7—C1	−179.54 (16)	C13—C14—C17—C18	144.64 (15)
C6—S1—C7—C5	−0.66 (12)	C15—C14—C17—C18	−37.13 (19)
N1—C6—C8—C9	−6.1 (2)	C13—C14—C17—C24	25.2 (2)
S1—C6—C8—C9	174.29 (12)	C15—C14—C17—C24	−156.60 (14)
N1—C6—C8—C12	172.08 (14)	C13—C14—C17—C23	−95.14 (17)
S1—C6—C8—C12	−7.5 (2)	C15—C14—C17—C23	83.09 (17)
C12—C8—C9—C10	−3.3 (2)	C14—C17—C18—C19	57.26 (18)
C6—C8—C9—C10	174.95 (14)	C24—C17—C18—C19	179.38 (14)
C8—C9—C10—C11	0.3 (2)	C23—C17—C18—C19	−62.00 (18)
C8—C9—C10—C13	−176.38 (15)	C15—N2—C19—C18	12.7 (2)
C12—O2—C11—C16	178.12 (13)	C20—N2—C19—C18	−164.74 (16)
C12—O2—C11—C10	−0.5 (2)	C17—C18—C19—N2	−46.8 (2)
C9—C10—C11—O2	1.6 (2)	C15—N2—C20—C21	18.0 (2)
C13—C10—C11—O2	178.49 (13)	C19—N2—C20—C21	−164.59 (16)
C9—C10—C11—C16	−176.89 (14)	N2—C20—C21—C22	−50.0 (2)
C13—C10—C11—C16	0.0 (2)	C11—C16—C22—C25	−85.78 (18)
C11—O2—C12—O1	178.43 (14)	C15—C16—C22—C25	88.70 (17)
C11—O2—C12—C8	−2.4 (2)	C11—C16—C22—C21	154.67 (14)
C9—C8—C12—O1	−176.73 (16)	C15—C16—C22—C21	−30.85 (18)
C6—C8—C12—O1	5.0 (2)	C11—C16—C22—C26	37.0 (2)
C9—C8—C12—O2	4.2 (2)	C15—C16—C22—C26	−148.48 (14)
C6—C8—C12—O2	−174.01 (13)	C20—C21—C22—C16	54.98 (18)
C11—C10—C13—C14	−1.4 (2)	C20—C21—C22—C25	−63.29 (18)
C9—C10—C13—C14	175.38 (15)	C20—C21—C22—C26	178.78 (14)
C10—C13—C14—C15	0.1 (2)		
